# Construction of a Nine-MicroRNA-Based Signature to Predict the Overall Survival of Esophageal Cancer Patients

**DOI:** 10.3389/fgene.2021.670405

**Published:** 2021-05-19

**Authors:** Xiaobin Zhang, Yi He, Haiyong Gu, Zhichao Liu, Bin Li, Yang Yang, Jie Hao, Rong Hua

**Affiliations:** ^1^Department of Thoracic Surgery, Shanghai Chest Hospital, Shanghai Jiao Tong University, Shanghai, China; ^2^Department of Cardiovascular Surgery, Shanghai Chest Hospital, Shanghai Jiao Tong University, Shanghai, China; ^3^Key Laboratory of Systems Biomedicine (Ministry of Education), Shanghai Center for Systems Biomedicine, Shanghai Jiao Tong University, Shanghai, China

**Keywords:** biomarkers, miRNAs, esophageal cancer, circulating miRNAs, overall survival time

## Abstract

**Background:**

Esophageal cancer (EC) is a common malignant tumor. MicroRNAs (miRNAs) play a key role in the occurrence and metastasis and are closely related to the prognosis of EC. Therefore, it will provide a powerful tool to predict the overall survival (OS) of EC patients based on miRNAs expression in EC tissues and blood samples.

**Methods:**

Five independent databases, TCGA, GSE106817, GSE113486, GSE122497, and GSE112264, were used to construct nine-miRna signature and nomograms for prognosis. The bioinformatics analysis was used to predict the enrichment pathways of targets.

**Results:**

A total of 132 overexpressed miRNAs and 23 suppressed miRNAs showed significant differential expression in both EC serum and tissue samples compared with normal samples. We also showed that nine miRNAs were related to the prognosis of EC. Higher levels of miR-15a-5p, miR-92a-3p, miR-92a-1-5p, miR-590-5p, miR-324-5p, miR-25-3p, miR-181b-5p, miR-421, and miR-93-5p were correlated to the shorter survival time in patients with EC. In addition, we constructed a risk prediction model based on the levels of nine differentially expressed miRNAs (DEMs) and found that the OS time of EC patients with high-risk score was shorter than that of EC patients with low-risk score. Furthermore, our results showed that the risk prediction scores of EC samples were higher than those of normal samples. Finally, the area under the curve (AUC) was used to analyze the risk characteristics of EC and normal controls. By calculating the AUC and the calibration curve, the RNA signature showed a good performance. Bioinformatics analysis showed that nine DEMs were associated with several crucial signaling, including p53, FoxO, PI3K-Akt, HIF-1, and TORC1 signaling. Finally, 14 messenger RNAs (mRNAs) were identified as hub targets of nine miRNAs, including BTRC, SIAH1, RNF138, CDC27, NEDD4L, MKRN1, RLIM, FBXO11, RNF34, MYLIP, FBXW7, RNF4, UBE3C, and RNF111. TCGA dataset validation showed that these hub targets were significantly differently expressed in EC tissues compared with normal samples.

**Conclusion:**

We have constructed maps and nomograms of nine-miRna risk signals associated with EC prognosis. Bioinformatics analysis revealed that nine DEMs were associated with several crucial signaling, including p53, FoxO, PI3K-Akt, HIF-1, and TORC1 signaling, in EC. We think that this study will provide clinicians with an effective decision-making tool.

## Introduction

Esophageal cancer (EC) is one of the most common cancers (Heitmiller, 2001; Eslick, 2009; Gu et al., 2020b). Traditionally, EC is divided into squamous cell carcinoma (ESCC) and adenocarcinoma (EAC) (Holmes and Vaughan, 2007). A total of 88% of EC are ESCCs (Holmes and Vaughan, 2007). Survival rates were low for both histologic types due to that most patients were diagnosed at a late stage. The 5-year survival rate worldwide is 15–25% (Uhlenhopp et al., 2020). According to previous reports, only one of the eight ECs has been identified in the early stages (Asombang et al., 2019). Most of the ECs are diagnosed after dysphagia, local tumor, and other symptoms (Ginex et al., 2013). Therefore, it is urgent to find early diagnostic markers for EC. Simulated staining endoscopy and magnifying endoscopy are the main methods for the early diagnosis of EC (Hoffman et al., 2017). However, it is worth noting that there is still a lack of effective cancer markers for the early diagnosis of EC. Of note, messenger RNA (mRNA), microRNA (miRNA), and long non-coding RNA (lncRNA) levels have also been found to correlate with tumor progression in ECs. For example, angiopoietin-like protein 2 is used as a new biomarker for EC (Ide et al., 2015). Improving the rate of early cancer screening is an effective method to reduce the mortality of EC.

MicroRNAs are composed of 18–25 nucleotides that regulate gene expression in many cancers (Ling et al., 2013). Some of these miRNAs are crucial for cancer progression and potentially related to diagnosis, assessment of therapeutic response, and prognostic prediction (Bertoli et al., 2015). According to previous reports, miRNAs play a crucial role in tumor cell growth and differentiation by modulating the expression of oncogenes or tumor suppressors (Frixa et al., 2015). Over the past decade, the potential functions of miRNAs as tumor markers have attracted considerable attention due to that miRNAs in tissues can distinguish cancer patients from healthy controls. Identification of miRNAs in individual body fluids opens new perspectives for non-invasive diagnosis, prognostic judgment, and prognosis prediction (Cortez et al., 2011). The role of circulating miRNAs (c-miRNAs) in tumorigenesis has attracted considerable attention, and the expression of multiple c-miRNAs had been reported to be differently expressed in many cancers, including but not limited to hepatocellular, prostate, and breast cancers (Cui et al., 2019). c-miRNAs can be detected in almost every cell type under various conditions, such as secretion, apoptosis, inflammation, and so on (Cui et al., 2019). In addition, the clinical importance of c-miRNAs in EC has been confirmed. For example, Shen et al. determined that the serum expression of miR-16-5p is correlated to ESCC (Reis et al., 2020). El Kirill et al. found that serum levels of miR-199a-3p are down-regulated in EC samples. Gu et al. (2018) found that serum miR-331-3p can predict the recurrence of EAC. However, there is no comprehensive analysis of the different expressions of c-miRNA in ECs.

In this study, we identified patients with differentially expressed miRNAs (DEMs) in EC serum. We also explored whether miRNAs are associated with the overall survival (OS) in patients with EC. Small RNA-based markers of DEMs and a new miRNA-based model were developed to predict the outcome in EC. This study provides a new biomarker for the early prognosis and treatment of EC.

## Materials and Methods

### Data Source and Preprocessing

Four datasets including GSE113486 (Usuba et al., 2019), GSE106817 (Yokoi et al., 2018), GSE122497 (Sudo et al., 2019), and GSE112264 (Urabe et al., 2019) were downloaded from GEO database and analyzed with the edgeR software package (Robinson et al., 2010). GSE106817 included serum miRNA profiles of 4,046 samples, which consist of 2,759 non-cancer controls, 88 EC samples, and 1,199 other solid cancer samples. GSE113486 included serum miRNA profiles of 972 samples, which consist of 100 non-cancer controls, 40 EC samples, and 832 other solid cancer samples. GSE122497 included serum miRNA profiles of 5,531 samples, which consist of 4,964 non-cancer controls and 567 EC samples. GSE112264 included serum miRNA profiles of 1,591 samples, which consist of 41 non-cancer controls, 50 EC samples, and 1,500 other solid cancer samples.

### Construction of Risk Scoring Formula

Lasso-CPHR analysis using the glmnet package of the R software was performed (Engebretsen and Bohlin, 2019). MiRNAs with | log_2_FC| ≥ 0 and a *P*-value < 0.01 were identified as significant DEMs in EC. The risk score was determined based on the regression coefficient of the miRNA level. The Risk Scoring Formula was calculated as Risk score = the sum of the coefficients × miRNA level using OncomiR database^1^.

### Evaluation of miRNA Characteristic

Kaplan–Meier survival analysis was applied to compare the OS between the high- and low-risk groups (Gu et al., 2020a). Survival ROC package was used to estimate the sensitivity and specificity of RNA signature based on the area under the curve (AUC) values.

### Functional Enrichment Analysis

Five databases, Starbase (Yang et al., 2011; Li et al., 2014), RNA22 (Loher and Rigoutsos, 2012), targetscan (Gu et al., 2020d), miRANda (Wang et al., 2014), and PicTAR (Krek et al., 2005), were applied to predict the targets of miRNAs. The GO and KEGG analyses were conducted using the David software (Shi et al., 2018; Gu et al., 2020c).

## Results

### Identification of DEMs in the Serum Samples of EC Patients

**FIGURE 1:**
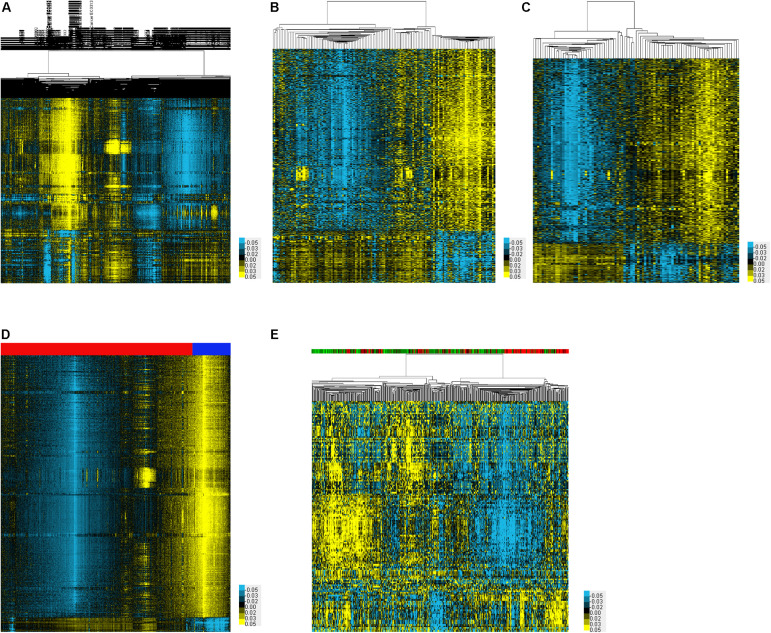
Identification of DEMs associated with EC. **(A–E)** The heatmap showed the DEMs in EC using GSE113486 **(A)**, GSE106817 **(B)**, GSE122497 **(C)**, GSE112264 **(D)**, and TCGA **(E)** databases, respectively.

In order to identify DEMs in the serum samples of EC, we analyzed four datasets including GSE113486, GSE106817, GSE122497, and GSE112264, which included 71 EC samples and 13 normal samples, 1,215 EC samples and 3,254 normal samples, 257 EC samples and 325 normal samples, and 2,576 EC samples and 2,654 normal samples, respectively. A total of 1,370, 1,326, 1,330, and 1,333 up-regulated miRNAs were identified in GSE113486 (Figure 1A), GSE106817 (Figure 1B), GSE122497 (Figure 1C), and GSE112264 (Figure 1D) databases, respectively. Meanwhile, 272, 262, 255, and 246 down-regulated miRNAs were identified in GSE113486 (Figure 1A), GSE106817 (Figure 1B), GSE122497 (Figure 1C), and GSE112264 (Figure 1D) databases, respectively. The expression levels of DEMs in these databases were visually displayed using a heatmap and volcano plot.

### Identification of DEMs in the Tissue Samples of EC Patients

**FIGURE 2:**
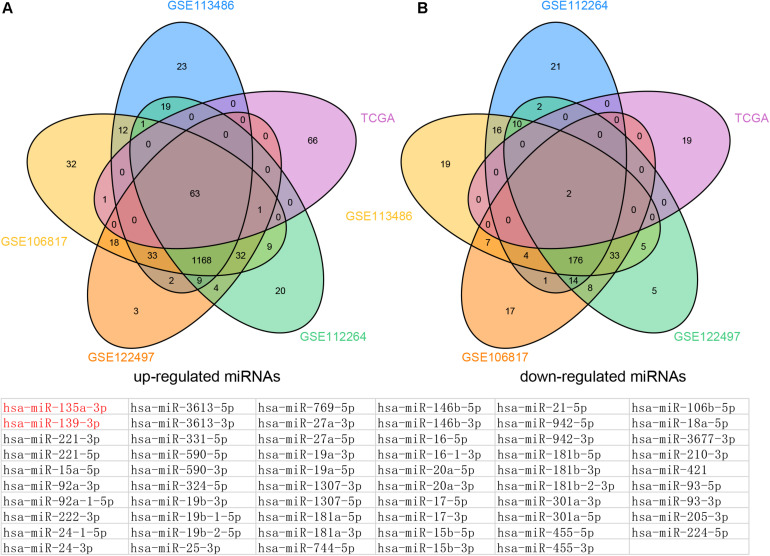
Venn map analysis of DEMs in Esophageal cancer. The common up-regulated **(A)** and down-regulated **(B)** miRNAs in EC using GSE113486, GSE106817, GSE122497, GSE112264, and TCGA databases, respectively.

In order to identify DEMs in the tissue samples of EC patients, we analyzed TCGA, which included 365 EC samples and 13 normal samples (Figure 1E). As shown in Figure 2, a total of 132 induced miRNAs (Figure 2A) and 23 suppressed miRNAs (Figure 2B) were observed in EC compared with normal tissues.

By combining these results, we finally identified 63 common up-regulated miRNAs (including miR-221-5p, miR-15a-5p, miR-92a-3p, miR-92a-1-5p, miR-222-3p, miR-24-1-5p, miR-24-3p, and miR-3613-5p) and two common down-regulated miRNAs (miR-135a-3p and miR-139-3p) in serum and tissue samples of EC compared with normal tissues.

### Identification of Survival Related to DEMs in EC

**FIGURE 3:**
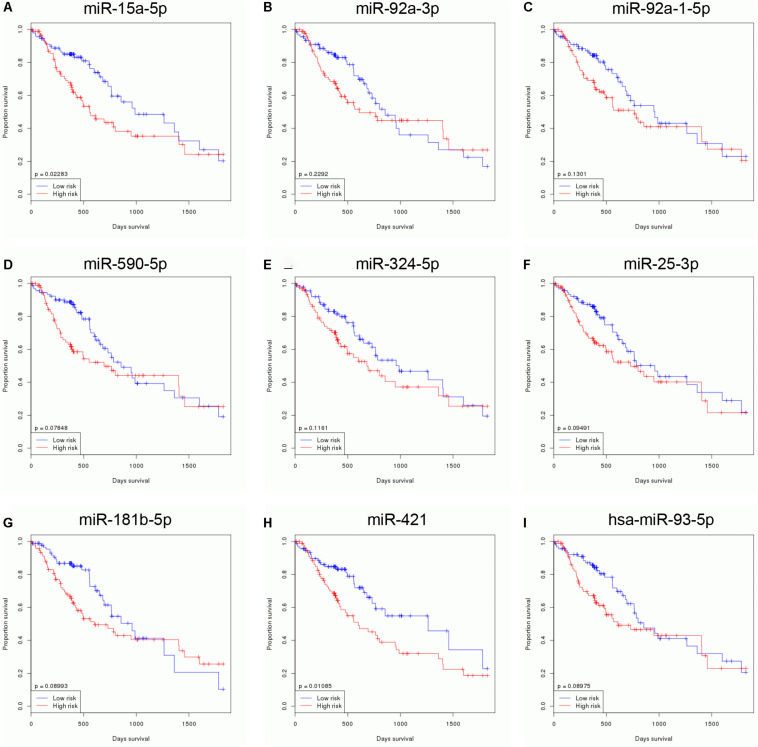
Identification of survival related DEMs in EC. Higher expression levels of miR-15a-5p **(A)**, miR-92a-3p **(B)**, miR-92a-1-5p **(C)**, miR-590-5p **(D)**, miR-324-5p **(E)**, miR-25-3p **(F)**, miR-181b-5p **(G)**, miR-421 **(H)**, and miR-93-5p **(I)** were correlated to the shorter survival time in patients with EC.

In order to identify survival related to DEMs in EC, we analyzed the correlation between 65 DEMs’ expression and OS time in EC using TCGA database. Our results showed that nine miRNAs were related to the prognosis of EC. Higher levels of miR-15a-5p (Figure 3A), miR-92a-3p (Figure 3B), miR-92a-1-5p (Figure 3C), miR-590-5p (Figure 3D), miR-324-5p (Figure 3E), miR-25-3p (Figure 3F), miR-181b-5p (Figure 3G), miR-421 (Figure 3H), and miR-93-5p (Figure 3I) were correlated to the shorter survival time in patients with EC. Very interestingly, we found that these miRNAs were significantly overexpressed in EC samples compared with normal tissues, suggesting that these miRNAs may serve as oncogenic miRNAs in EC (Figures 1A–E).

### Construction of a Risk Signature

**FIGURE 4:**
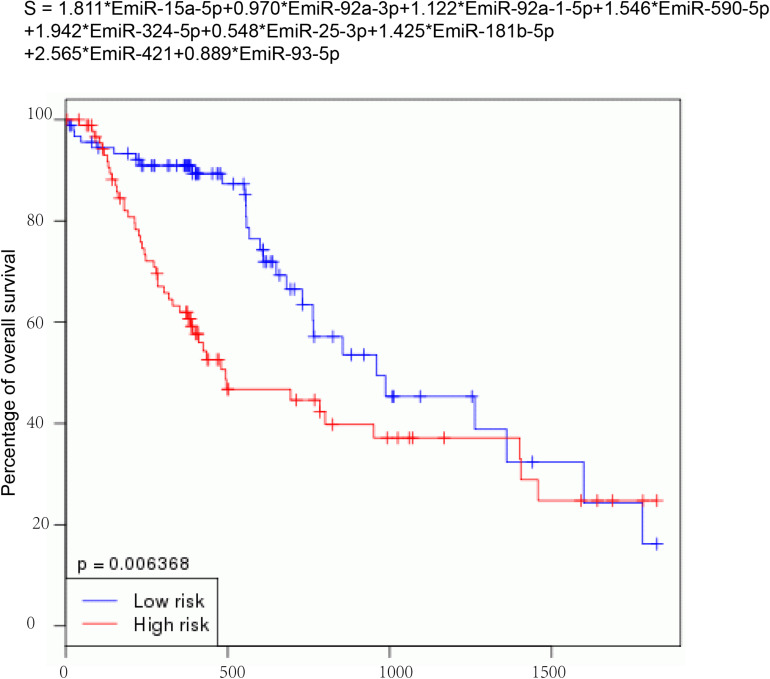
Construction of a risk signature. Nine DEMs were used to develop a risk signature: Risk score = 1.811*EmiR-15a-5p + 0.970*EmiR-92a-3p + 1.122*EmiR-92a-1-5p + 1.546*EmiR-590-5p + 1.942*EmiR-324-5p + 0.548*EmiR-25-3p + 1.425*EmiR-181b-5p + 2.565*EmiR-421 + 0.889*EmiR-93-5p.

Then, nine DEMs were used to develop a risk signature: Risk score = 1.811^∗^EmiR-15a-5p + 0.970^∗^EmiR-92a-3p + 1.122^∗^EmiR-92a-1-5p + 1.546^∗^EmiR-590-5p + 1.942^∗^EmiR-324-5p + 0.548^∗^EmiR-25-3p + 1.425^∗^EmiR-181b-5p + 2.565^∗^EmiR-421 + 0.889^∗^EmiR-93-5p (Figure 4). We observed that the EC with high-risk score value had shorter OS time than that with low-risk score value (*P*-value = 0.006368) (Figure 4).

### Estimation of the Reliability of the Risk Signature

**FIGURE 5:**
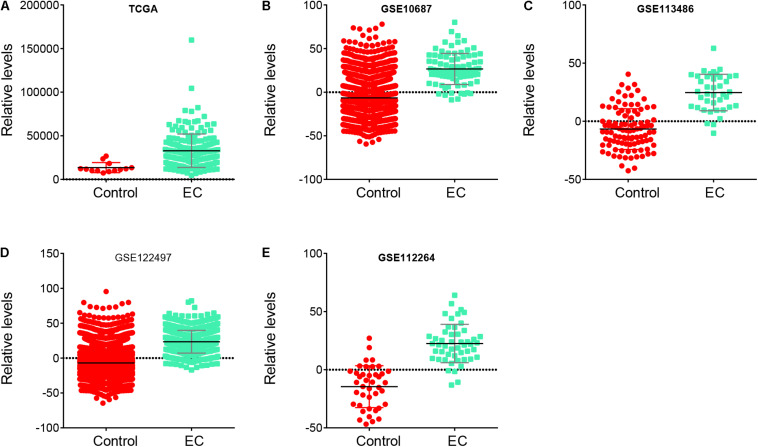
Risk signature score in EC samples was higher than that in normal samples. **(A–E)** The risk signature score in EC samples was higher than that in normal samples in TCGA **(A)**, GSE106817 **(B)**, GSE113486 **(C)**, GSE122497 **(D)**, and GSE112264 **(E)** databases.

To further confirm the reliability of the risk signature in EC, we analyzed the risk signature score between normal samples and EC samples in these databases, including TCGA, GSE106817, GSE113486, GSE122497, and GSE112264. Our results showed that the risk signature score in EC was higher than that in normal samples in TCGA (Figure 5A), GSE106817 (Figure 5B), GSE113486 (Figure 5C), GSE122497 (Figure 5D), and GSE112264 (Figure 5E) databases.

**FIGURE 6:**
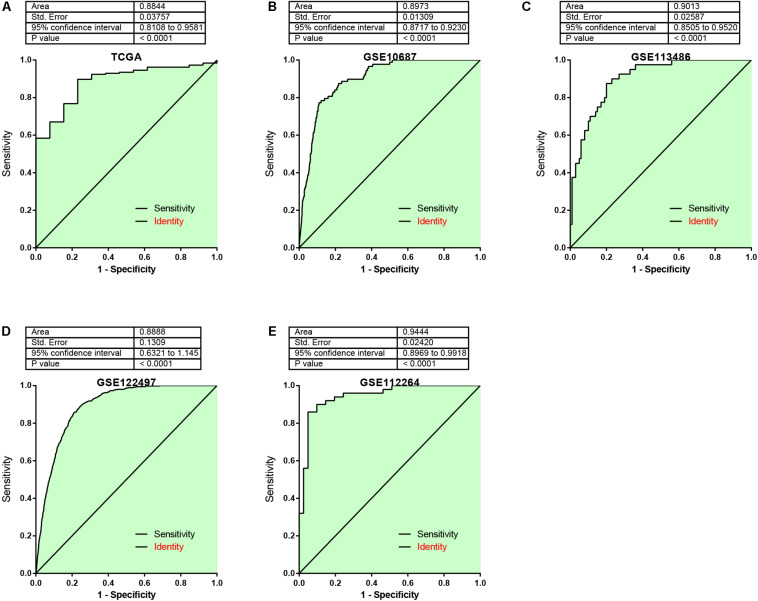
The AUC analysis of the risk signature for distinguishing EC from normal samples was analyzed. **(A–E)** ROC curve analyses for the risk signature were 0.8844, 0.897, 0.9013, 0.888, and 0.9444 by analyzing TCGA **(A)**, GSE106817 **(B)**, GSE113486 **(C)**, GSE122497 **(D)**, and GSE112264 **(E)** cohorts, respectively.

In addition, the AUC analysis of the risk signature for distinguishing EC from normal samples was analyzed. In TCGA (Figure 6A), GSE106817 (Figure 6B), GSE113486 (Figure 6C), GSE122497 (Figure 6D), and GSE112264 (Figure 6E) cohorts, the receiver operating characteristic (ROC) curve analyses for the risk signature were 0.8844, 0.897, 0.9013, 0.888, and 0.9444, respectively, indicating considerable accuracy.

### Bioinformatics Analysis of DEMs

**FIGURE 7:**
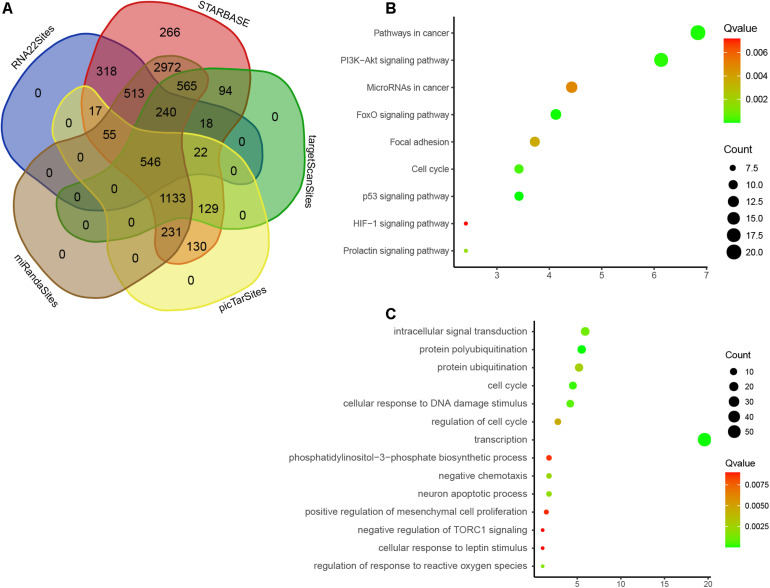
Bioinformatics analysis of DEMs. **(A)** Venn map analysis of potential targets of nine DEMs by using five online databases, Starbase, RNA22, targetscan, miRANda, and PicTAR databases. **(B)** GO analysis of DEMs in EC. **(C)** KEGG enrichment analyses of DEMs in EC.

We used five databases to predict the roles of nine DEMs and detected 14,973 target genes, 546 of which were overlapped (Figure 7A). Therefore, these overlapping genes that may be regulated by nine DEMs were analyzed by GO and KEGG enrichment. We observed that nine DEMs are associated with p53 signaling, FoxO signaling, PI3K-Akt signaling, cell cycle, prolactin signaling, focal adhesion, miRNAs in cancer, and HIF-1 signaling (Figure 7B). GO analysis showed that nine DEMs were related to regulate protein polyubiquitination, transcription, cell cycle, DNA damage stimulus, intracellular signal transduction, response to reactive oxygen species, neuron apoptotic process, protein ubiquitination, cell cycle, phosphatidylinositol-3-phosphate biosynthetic process, mesenchymal cell proliferation, cellular response to leptin stimulus, and TORC1 signaling (Figure 7C).

### Identification and Validation of Hub Targets of Nine miRNAs in EC

**FIGURE 8:**
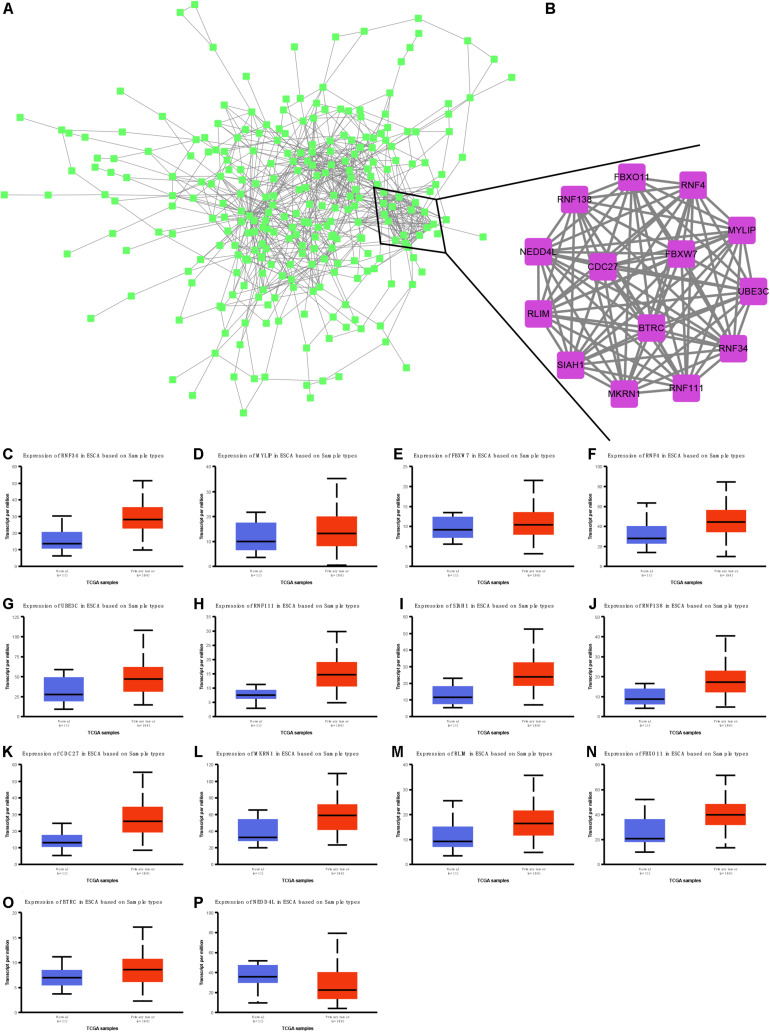
Identification and validation of hub targets of nine miRNAs in EC. **(A)** Protein–protein interaction network analysis to identify hub targets of nine miRNAs. **(B)** Ten mRNAs were identified as hub targets, including BTRC, SIAH1, RNF138, CDC27, NEDD4L, MKRN1, RLIM, FBXO11, RNF34, MYLIP, FBXW7, RNF4, UBE3C, and RNF111. **(C–P)** RNF34 **(C)**, MYLIP **(D)**, FBXW7 **(E)**, RNF4 **(F)**, UBE3C **(G)**, RNF111 **(H)**, SIAH1 **(I)**, RNF138 **(J)**, CDC27 **(K)**, MKRN1 **(L)**, RLIM **(M)**, FBXO11 **(N)**, BTRC **(O)**, and NEDD4L **(P)** were remarkably suppressed in EC tissues compared with normal samples.

We next performed protein–protein interaction network analysis to identify hub targets of nine miRNAs. A total of 284 nodes and 785 edges were included in this network (Figure 8A). Fourteen mRNAs were identified as hub targets, including BTRC, SIAH1, RNF138, CDC27, NEDD4L, MKRN1, RLIM, FBXO11, RNF34, MYLIP, FBXW7, RNF4, UBE3C, and RNF111 (Figure 8B). We also analyzed the correlation between these mRNAs and clinical parameters in EC using TCGA database. Our results showed that RNF34 (Figure 8C), MYLIP (Figure 8D), FBXW7 (Figure 8E), RNF4 (Figure 8F), UBE3C (Figure 8G), RNF111 (Figure 8H), SIAH1 (Figure 8I), RNF138 (Figure 8J), CDC27 (Figure 8K), MKRN1 (Figure 8L), RLIM (Figure 8M), FBXO11 (Figure 8N), and BTRC (Figure 8O) were significantly up-regulated; however, NEDD4L (Figure 8P) was remarkably suppressed in EC tissues compared with normal samples.

## Discussion

Many reports have shown that miRNAs are related to many pathological processes of EC (Sharma and Sharma, 2015), but there are no miRNAs that can predict the OS in EC patients. Moreover, the results of previous studies were obtained based on limited sample counts and limited datasets. In this study, we used five independent databases, including TCGA, GSE106817, GSE113486, GSE122497, and GSE112264, to identify DEMs in EC. More than 12,500 samples were included in this study. Furthermore, we analyzed miRNA expression levels between normal and EC samples to systematically determine the correlation between miRNA and clinical characteristics and prognosis, as well as OS time. We finally identified 63 common up-regulated miRNAs (such as miR-221-5p, miR-15a-5p, miR-92a-3p, miR-92a-1-5p, miR-222-3p, Has-miR-24-1-5p, miR-24-3p, and miR-3613-5p) and two common down-regulated miRNAs (miR-135a-3p and miR-139-3p) in serum and tumor tissues in EC patients compared with normal tissues. Further analysis showed that nine miRNAs were related to the prognosis of EC. Higher levels of miR-15a-5p, miR-92a-3p, miR-92a-1-5p, miR-590-5p, miR-324-5p, miR-25-3p, miR-181b-5p, miR-421, and miR-93-5p were correlated to the shorter survival time in patients with EC.

Over the past decade, “liquid biopsies” have shown potential to be novel biomarkers for cancer prognosis (Schlange and Pantel, 2016; Marrugo-Ramirez et al., 2018). Circulating tumor cells (CTCs)/DNA (ctDNA), cell-free miRNA (cfmiRna), tumor-induced platelets, and extracellular vesicles are relatively new blood-based biomarkers that can be used in biomarkers development (Lowes et al., 2016). These biomarkers have been shown to have the capacities to detect solid tumors and to provide prognostic and predictive information about the disease in multiple cancer types. In the present study, we observe that miR-15a-5p, miR-92a-3p, min-92a-1-5p, has-miR-590-5p, miR-181b-5p, Hsa-miR-421, and miR-93-5p were related to shorter OS in EC. In addition, we constructed a risk prediction model based on the expression levels of miR-15a-5p, miR-92a-3p, miR-92a-1-5p, miR-590-5p, miR-324-5p, miR-25-3p, miR-181b-5p, miR-421, and miR-93-5p and found that the OS time of EC patients with high-risk score is shorter than that of EC patients with low-risk score. Furthermore, our results showed that in TCGA, GSE106817, GSE113486, GSE122497, and GSE112264, the risk prediction scores of EC samples were higher than those of normal samples. Finally, AUC was used to analyze the risk characteristics of EC and normal controls. The AUC values of EC and normal subjects were 0.8844, 0.897, 0.9013, 0.888, and 0.9444, respectively, and suggested that this miRNA signature is a new biomarker for the prediction and diagnosis of EC.

Remarkably, several members of this RNA signature have been shown to be key regulators of human cancer. For example, miR-25 is reported to be a crucial regulator of cancers and non-cancer diseases (Caiazza and Mallardo, 2016). In tumors, miR-25 is a well-described oncogenic miRNA in a variety of cancers (Caiazza and Mallardo, 2016), including brain, lung, breast, ovarian, esophageal, stomach, colorectal, and liver cancers. Many miR-25-targeted mRNAs had been revealed to modulate cell growth, migration, oxidative stress, inflammation, and other biological processes (Caiazza and Mallardo, 2016). A previous study reported that miR-25 was a new marker for the diagnosis and monitoring of ESCC by analyzing plasma miRNA (Jia et al., 2017). MiR-25 overexpression is associated with lymph node metastasis. MiR-25 promotes metastasis of ESCC by targeting Fbxw7 and E-cadherin signaling pathways (Hua et al., 2017). miR-25-3p targeted Pten through PI3K/Akt pathway to regulate EC migration, invasion, and apoptosis (Zhang et al., 2020). MiR-93-5p and miR-324-5p are also reported to be an oncogene in many cancers, such as non-small-cell lung carcinoma, hepatocellular carcinoma, prostate cancer, and cervical cancer (Wu et al., 2014; Xiang et al., 2017; Yang et al., 2019; Vila-Navarro et al., 2020). In the esophagus, the importance and clinical significance of miR-93-5p are also illustrated. For example, exogenous miR-93-5p promotes EC cell proliferation and communication by targeting Pten. MiR-324-5p may promote the progression of thyroid, oropharyngeal, pancreatic, and lung cancer cells, polarized M2 macrophages in colon cancer, and modulated the microenvironment in thyroid cancer (Wan et al., 2020). However, the functions of miR-324-5p in EC remain unclear.

It is interesting that these miRNAs were related to regulate protein polyubiquitination, transcription, cell cycle, DNA damage stimulus, cell cycle, phosphatidylinositol-3-phosphate biosynthetic process, and mesenchymal cell proliferation by using bioinformatics analysis. Of note, in this study, we identified that nine DEMs are associated with several crucial signaling, including p53, FoxO, PI3K-Akt, HIF-1, and TORC1 signaling. These signaling had crucial roles in cancer cells. For example, PI3K/AKT pathway was activated in EC samples (Javadinia et al., 2018). Using specific inhibitors targeting this pathway significantly inhibited cell proliferation, enhancing apoptosis in EC cells (Shi et al., 2019). HIF-1, a transcription factor, plays a major role in the regulation of angiogenesis, glucose transport, and erythropoiesis. Previous studies showed that HIF-1α expression was higher in EC samples and significantly related to lower recurrence-free survival. Finally, 14 mRNAs were identified as hub targets of nine miRNAs, including BTRC, SIAH1, RNF138, CDC27, NEDD4L, MKRN1, RLIM, FBXO11, RNF34, MYLIP, FBXW7, RNF4, UBE3C, and RNF111. TCGA dataset validation showed that these hub targets were significantly differently expressed in EC tissues compared with normal samples. These studies, together with our findings, further demonstrate the potential functional importance of these miRNAs in endothelial cells.

## Conclusion

In this study, we used five independent databases to analyze miRNA levels and clinical stages in EC. We have constructed maps and nomograms of nine-miRna risk signals associated with EC prognosis. Bioinformatics analysis revealed that nine DEMs are associated with several crucial signaling, including p53, FoxO, PI3K-Akt, HIF-1, and TORC1 signaling, in EC. We think that this study will provide clinicians with an effective decision-making tool.

## Data Availability Statement

The original contributions presented in the study are included in the article/supplementary material, further inquiries can be directed to the corresponding authors.

## Author Contributions

RH and JH contributed to the conception of the study and designed and organized the study. XZ and YH collected and analyzed the data. HG provided the methodology. All authors wrote the manuscript.

## Conflict of Interest

The authors declare that the research was conducted in the absence of any commercial or financial relationships that could be construed as a potential conflict of interest.

## Footnotes

1 http://www.oncomir.org/oncomir/index.html
